# Full-course resection control strategy in glioma surgery using both intraoperative ultrasound and intraoperative MRI

**DOI:** 10.3389/fonc.2022.955807

**Published:** 2022-08-25

**Authors:** Yuanzheng Hou, Ye Li, Qiongge Li, Yang Yu, Jie Tang

**Affiliations:** ^1^ Department of Neurosurgery, Xuanwu Hospital, Capital Medical University, Beijing, China; ^2^ Department of Radiology, Xuanwu Hospital, Capital Medical University, Beijing, China

**Keywords:** intraoperative ultrasound, intraoperative MRI, navigation, glioma, neurosurgery

## Abstract

**Background:**

Intraoperative ultrasound(iUS) and intraoperative MRI (iMRI) are effective ways to perform resection control during glioma surgery. However, most published studies employed only one modality. Few studies have used both during surgery. How to combine these two techniques reasonably, and what advantages they could have for glioma surgery are still open questions.

**Methods:**

We retrospectively reviewed a series of consecutive patients who underwent initial surgical treatment of supratentorial gliomas in our center. We utilized a full-course resection control strategy to combine iUS and iMRI: IUS for pre-resection assessment and intermediate resection control; iMRI for final resection control. The basic patient characteristics, surgical results, iMRI/iUS findings, and their impacts on surgical procedures were evaluated and reported.

**Results:**

A total of 40 patients were included. The extent of resection was 95.43 ± 10.37%, and the gross total resection rate was 72.5%. The median residual tumor size was 6.39 cm^3^ (range 1.06–16.23 cm3). 5% (2/40) of patients had permanent neurological deficits after surgery. 17.5% (7/40) of patients received further resection after the first iMRI scan, resulting in four (10%) more patients achieving gross total resection. The number of iMRI scans per patient was 1.18 ± 0.38. The surgical time was 4.5 ± 3.6 hours. The pre-resection iUS scan revealed that an average of 3.8 borders of the tumor were beside sulci in 75% (30/40) patients. Intermediate resection control was utilized in 67.5% (27/40) of patients. In 37.5% (15/40) of patients, the surgical procedures were changed intraoperatively based on the iUS findings. Compared with iMRI, the sensitivity and specificity of iUS for residual tumors were 46% and 96%, respectively.

**Conclusion:**

The full-course resection control strategy by combining iUS and iMRI could be successfully implemented with good surgical results in initial glioma surgeries. This strategy might stabilize resection control quality and provide the surgeon with more intraoperative information to tailor the surgical strategy. Compared with iMRI-assisted glioma surgery, this strategy might improve efficiency by reducing the number of iMRI scans and shortening surgery time.

## Introduction

Increasing the extent of resection (EOR) significantly improves overall survival (OS) in low-grade glioma (LGG) and High-grade glioma (HGG) (1, 2). The cut-off beneficial EOR is 90% for LGG (2), and 93% for the glioblastoma (3). Even if EOR exceeds 90%, every 5% increment can still significantly improve OS (1). Hence, the primary goal of the initial surgical treatment of glioma is to obtain the maximal EOR safely (4, 5). To achieve this goal, it is essential to assess the resection process during surgery precisely. However, surgeon estimation based on visualization is prone to error (6, 7). Only 30.4% of gross total resection (GTR) estimated by the surgeon could be confirmed by MRI (6). Therefore, intraoperative imaging techniques such as intraoperative ultrasound (iUS) and intraoperative MRI (iMRI) were introduced into glioma surgery for resection control during surgery.

IUS has been used in neurosurgery since the 1980s (8). Many studies have demonstrated its benefits in glioma surgery (8–10). The advantage of iUS is that it allows nearly real-time imaging without interfering with the surgical workflow (11). The major drawback is its steep learning curve and artifacts (12). The iMRI system was introduced into neurosurgery in the 1990s. Modern high-field iMRI systems can generate high-quality intraoperative imaging. A growing body of evidence showed that iMRI increased EOR and the rate of GTR and improved progression-free survival (PFS) and OS after glioma surgery (4, 5). However, iMRI has a significant disadvantage: it is time-consuming and interrupts the operation’s progress. Because of this, most centers preferred to perform iMRI scans only at the end of the resection process (13–15).

The characteristics of iUS and iMRI make it possible to combine them during glioma surgery. In the initial assessment, iUS has high sensitivity and specificity for detecting tumor boundaries (95% for glioblastoma (16), and are 72%–86% and 75%–100%, respectively, for LGG (17)). Intermediate iUS scans can be performed during resection with acceptable sensitivity (87%) (16). At the end of the resection, its sensitivity might drop to as low as 26% (12, 16, 18). With iUS for monitoring the resection process and iMRI for final resection control, the whole tumor resection process can be monitored by capitalizing on the advantages of both imaging modalities. This full-course resection control strategy might help surgeons make more informed decisions and perform better resection control.

However, most published studies employ only one modality during surgery or just use iMRI to verify the accuracy of iUS. Recently, Bastos et al. reported their experiences of using 3D iUS and iMRI together in 23 patients with glioma (19). Their study demonstrated the feasibility and benefits of this approach. In this study, we reported our experiences of using the full-course resection control strategy during glioma surgery and discussed the advantages and limitations of this strategy.

## Materials and methods

### Patient population

The supratentorial glioma patients who underwent surgery by the surgical team between July 2020 and June 2021 were retrospectively analyzed. The glioma diagnosis was confirmed by the final histopathological findings in accordance with the WHO guidelines. The patients who received initial glioma surgeries were included. We didn’t include recurrent cases because the iUS image quality varied in these patients (20).

### Training Level of the surgical team

The surgical team consisted of three neurosurgeons and two radiologists. Three neurosurgeons had completed two months of iUS training, followed by 43 cases of experience. Two of the three neurosurgeons had ten years of surgical experience using iMRI and navigation systems. Another neurosurgeon and both radiologists had two years of iMRI experience.

### Equipment

**Figure 1:**
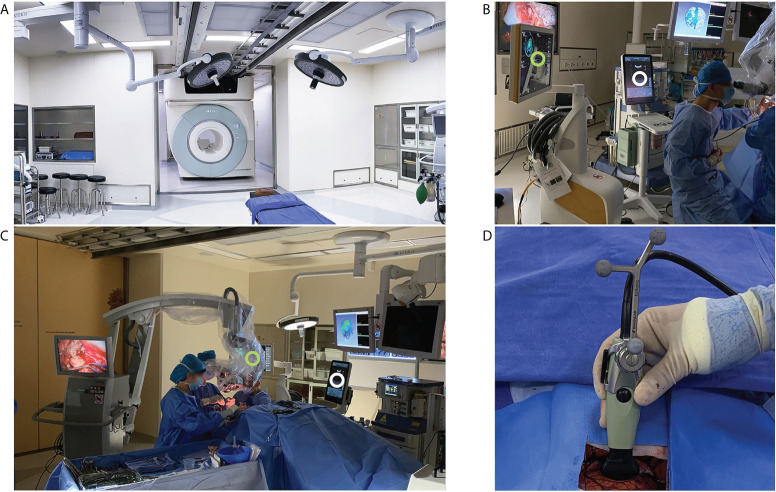
iMRI and iUS systems used in this study. **(A)** the iMRI system integrated a movable 3.0T MRI scanner. With the shielded door open, the scanner could move into the operating room to perform an iMRI scan. **(B)** iUS (white circle) and the navigation system (green circle) were registered during surgery to produce 3D iUS and co-planar MRI images. **(C)** During surgery, the shielded door was closed to isolate the magnetic field. iUS (white circle) and navigation system (Green circle) were placed on the left side of the surgeon. **(D)** iUS was registered with the navigation system by attaching a rigid reference frame to the transducer.

An iMRI hybrid operating suite (IMRIS, Minnetonka, USA) was used (**Figure 1A**). This system integrated a movable 3.0T MRI scanner (Magnetom Verio, Siemens Healthcare, Erlangen, Germany) and neuronavigation system (Curve^®^, Brainlab, Feldkirchen, Germany) running the iPlan 3.0 neuronavigation software (Brainlab, Feldkirchen, Germany) (**Figures 1B, C**). IUS was done using the BK iUS system (BK 3000, BK Medical, Herlev, Denmark) (**Figures 1B, C**). The craniotomy transducer (8862, BK Medical, Herlev, Denmark) and burr-hole transducer (8863, BK Medical, Herlev, Denmark) were used. The craniotomy transducer had a 10-× 29-mm contact surface 5- to 13-MHz frequency range. The burr-hole transducer had a 10-× 8.6-mm contact surface 5- to 11-MHz frequency range. The iUS was registered with the navigation system by rigidly attaching a reference frame (IGSonic, Brainlab, Feldkirchen, Germany) to the ultrasound transducer (**Figure 1D**). The navigation system could track the transducer, generate co-planar images of ultrasound and MR images, and perform 3D iUS scans.

### Preoperative MRI, multimodule functional navigation, and electrophysiological monitoring

Preoperatively, some or all of the following sequences were routinely scanned based on the tumor position (1): T1-weighted magnetization‐prepared rapid gradient‐echo (MPRAGE) sequence with and without gadolinium‐diethylenetriamine Penta‐acetic acid (Gd-DTPA) (2), axial T2 and T2-weighted fluid-attenuated inversion recovery (FLAIR) (3), DTI (4), functional MRI. All image sequences were imported into the iPlan neuronavigation software and co-registered before further processing. The tumor was segmented and reconstructed as a 3D model based on the T1 MPRAGE (with enhancement) or T2-weighted FLAIR (without enhancement) images. The software automatically calculated the tumor volume after segmentation. The pyramidal tract, language-related tracts (arcuated fasciculus, superior longitudinal fasciculus, inferior longitudinal fasciculus, and uncinate fasciculus), and optic radiation were reconstructed based on the DTI data using a deterministic algorithm. Functional MRI data were preprocessed using the SPM package. The results were imported into iPlan navigation software to reconstruct the Brocca area, Wernicke area, or foot, hand, and tongue motion areas. All data were exported into the navigation system to perform multimodule functional navigation during surgery. If the tumor involved the abovementioned eloquent structures, the awake craniotomy and cortical and sub-cortical mapping techniques were utilized following the strategies recommended in the literature. The positive mapping points were marked in the navigation system.

### IUS and iMRI scan

The published methods were followed to ensure adequate iUS image quality (21). The iUS was routinely registered with the navigation system during surgery (**Figures 1B, C**). The iUS images and reconstructed co-planar iMRI images were demonstrated simultaneously to facilitate the interpretation of the iUS images. 3D iUS images were acquired when necessary. The burr-hole transducer was used when the surgical corridors were too small, and the craniotomy transducer could not be inserted into the resection cavity (22).

**Figure 2:**
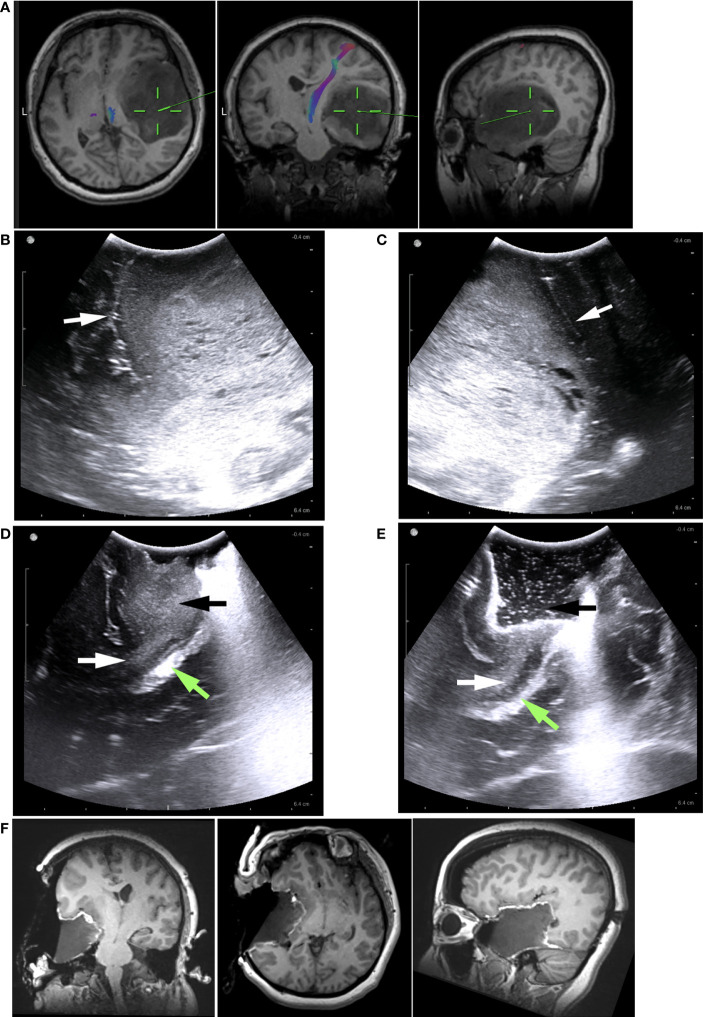
Illustrative case 1. **(A)** The preoperative MRI images revealed a low-grade glioma, predominantly in the right temporal lobe. It was difficult to determine how the tumor is related to the Sylvian fissure, insular lobe, or basal ganglia based on MRI images. **(B)** Pre-resection iUS image showing the tumor’s hyperechoic signal. Sylvian fissure (white arrow) and insular cortex were clearly visible on the iUS images. The insular cortex was not infiltrated by the tumor. The Sylvian fissure marked the upper border of tumor **(C)** In another pre-resection iUS image, the posterior margin was marked by a sulcus (white arrow). **(D)** Following partial removal of the tumor, the temporal stem (white arrows) was visible on the iUS image. There was no sign that the tumor (black arrow) had grown into basal ganglia through the temporal stem. The Green arrow indicated the ventricle. **(E)** Intermediated iUS revealed that the tumor was further removed along the temporal stem (white arrow). The Green arrow indicated the ventricle. **(F)** In iMRI images, the tumor was successfully removed along the Sylvian fissure and temporal stem. The insular cortex and basal ganglia were preserved.

**Figure 3:**
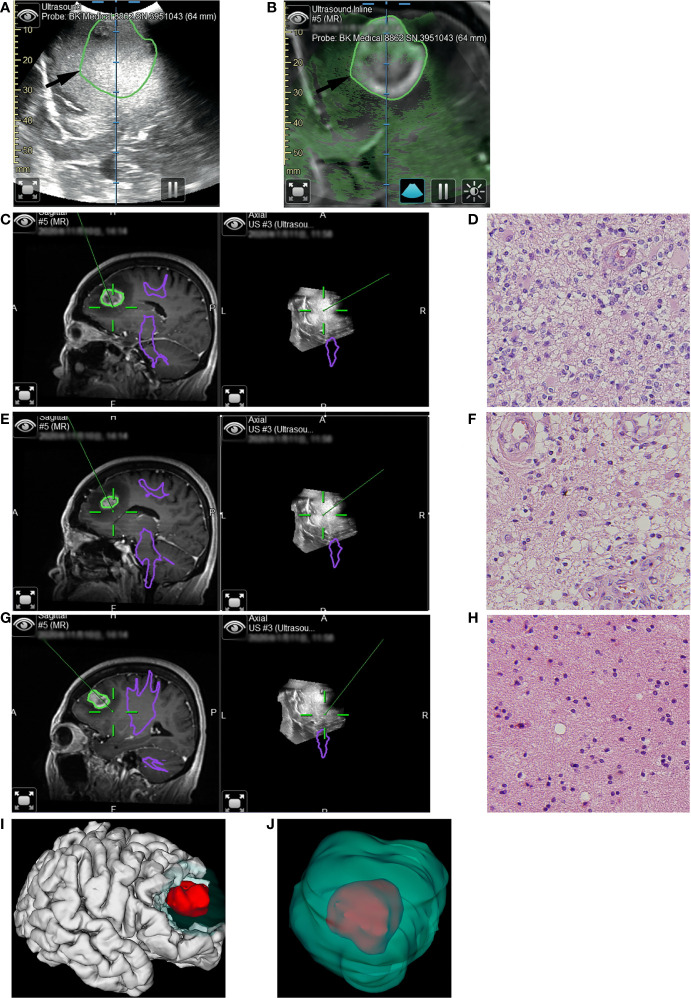
Illustrative case 2. **(A)** The pre-resection iUS image was registered with MRI using navigation. **(B)** Overlay of iUS image on coplanar MRI images. The black arrows indicated the border of the enhanced tumor on T1+C images. Compared to the enhanced tumor, the hyperechoic area was larger. **(C)** The first tissue sample was taken during resection of the enhanced tumor. On the co-planar 3D iUS, this part was hyperechoic. **(D)** Hematoxylin and eosin (H&E) stain (×40) showed marked hypercellularity, cellular and nuclear atypia, increased mitotic figures, and hypervascularity. **(E)** The second tissue sample was taken outside the enhanced lesion, where MRI showed an abnormally long T1 signal. Co-planar 3D iUS showed a similar signal at this site as the enhanced part. **(F)** H&E stain (×40) demonstrated the same characteristics as image D, revealing moderate infiltrative growth of tumor cells. **(G)** the third sample was taken after all tissue with abnormal MRI signals had been removed. At this site, the iUS were still hyperechoic but slightly lower than the previous site. **(H)** H&E stain (×40) demonstrated the microstructure of white matter, with mild gliosis and nuclear atypia. **(I, J)** 3D reconstruction based on iMRI images. The removed tissues with hyperechoic signals were segmented as green volumes, which measured 60.6cm^3^. As a comparison, the enhanced part was segmented as red volume, which was only 8.1cm^3.^.

We adopted the full-course resection control strategy to perform iUS and iMRI scans. The first iUS scan was done following dural opening (**Figures 2B, C**, **3A**). The view depth was set deep to make the field of view as big as possible to obtain an overview. The echo strength, position of the tumor, and surrounding edema were confirmed, and the tumor’s orientation and depth were decided accordingly (**Figure 3A**). The view depth was then made shallower to view the deep structures, such as the ventricles, Sylvian fissure, tentorium, and falx (**Figures 2B**, **3A**). These structures acted as the markers of the margins of the tumor. Next, the view depth was adjusted to just beneath the brain surface. The images were zoomed in to depict the topology of the gyri and sulci surrounding the tumor. The relationship between the tumor margins and the nearest sulci was carefully evaluated. These sulci were regarded as the landmarks for the tumor margins (**Figure 2C**).

The surgeon performed serial iUS scans at intermediate stages to monitor the tumor resection process (**Figures 2D, E**). Two basic rules about the intermediate iUS scan were made for the surgical team: a. If not necessary, intermediate iUS scans could be omitted; b. If there was any uncertainty regarding the resection process, attempt to resolve it with iUS. The surgical strategy might be tailored based on the intermediate iUS findings.

When the surgeon considered the intended tumor removal complete, a final iUS scan was performed to detect an unexpected residual tumor (**Figure 2F**). The final iUS results were used for comparison with the iMRI findings.

iMRI was used for the final resection control. T1 MPRAGE with and without Gd-DTPA and axial T2 and FLAIR sequence images were scanned to detect the residual tumor. If an unexpected residual tumor was detected, the iMRI sequences were imported into iPlan software and co-registered with the preoperative MRI sequences. The residual tumor was then segmented and reconstructed in the navigation system. The residual tumor volume was recorded.

The surgeon performed further tumor resection assisted by the updated navigation data. Additional iMRI scans were routinely performed if further resection was finished.

### Data collection

The demographic data and pathological results were collected from the patient records by the first and second authors. The tumor characteristics were recorded by reviewing the preoperative MR images. The third and fourth authors volumetrically analyzed the tumors and residual tumors using MRI and iMRI data. For low-grade gliomas (LGG), tumor volumes were calculated by manual segmentation on FLAIR or T2 axial slices. For high-grade gliomas (HGG), a similar calculation was made using the volume of contrast-enhanced tissue on T1 MPRAGE with Gd-DTPA. The EOR was calculated as (preoperative tumor volume − postoperative tumor volume)/preoperative tumor volume. GTR means that EOR is equal to 100%. The rate of GTR was calculated.

The first and second authors reviewed the long-term outcome three months after surgery by performing telephone interviews. The Karnofsky performance scale (KPS) was calculated before surgery and three months after surgery.

iMRI images were analyzed by the third and fourth authors. The residual tumor volume at the first iMRI scan was calculated. The EOR, according to the first iMRI scan, was calculated using the same formula. The number of iMRI scans during each surgery was also recorded.

The number of iUS scans and the iUS findings was analyzed postoperatively by the first and senior author. The tumor margins that could be marked by sulci were recorded using the following method. The tumor margins were simplified into a box without a lid with five borders (anterior, posterior, medial, lateral, and deep) regarding the surgeon’s angle of view. The number of borders for which a sulcus could be used as a marker was noted. The number of such borders was zero for deep tumors located away from the brain surface (such as a glioma situated in the basal ganglia area). The number of patients in which the intermediate resection control strategy was utilized was calculated. The number of cases in which the surgical procedure was changed intraoperatively in accordance with intermediate iUS scans was calculated. The results of final control iUS for detecting residual tumors were dichotomized into “yes” and “no” during surgery and recorded. A “yes” result means that the surgical team confirmed the presence of residual tumor using iUS images. If the surgical team could not detect any residual tumor or could not make a congruent decision because of artifacts or low image quality, the result was categorized as “no.” The sensitivity and specificity of the iUS were calculated compared to the first iMRI scan results—the surgical time after anesthetization was recorded and analyzed by the first authors.

### Statistical analysis

Frequency distributions and summary statistics were calculated with SPSS 20.0 (Lead Technologies, Inc., Charlotte, NC, USA).

## Results

A total of 40 patients were included in this study. The clinical characteristics and primary surgical results are summarized in **Table 1**. The detailed iUS and iMRI findings for each patient are listed in **Table 2**.

**Table 1:** Pre- and intra-operative data and surgical results.

**N**	40
Age (mean [SD])	48.28 (12.66)
**Gender**	
Male (%)	18 (45%)
Female (%)	22 (55%)
**Pathological results**	
LGG (%)	9 (22.5%)
HGG (%)	31 (77.5%)
**Localization**	
Non-eloquent (%)	15 (37.5%)
Eloquent (%)	25 (62.5%)
**Tumor volume cm^3^ (mean [SD])**	61.64 (44.53)
**Preoperative KPS (mean [SD])**	90.25 (12.08)
**iMRI scan**	
Times per patient (mean [SD])	1.18 (0.38)
Found Residual tumor in first iMRI scan (%)	15 (37.5%)
EOR based first iMRI results % (mean [SD])	93.63% (11.32)
RTV in first iMRi scan cm^3^ (mean [SD])	3.24 (5.57)
**Final EOR % (mean [SD])**	95.43 (10.37)
**Final Rate of GTR (%)**	29 (72.5%)
**Postoperative KPS (Mean [SD])**	85.0 (23.75)

LGG, low-grade glioma; HGG, high-grade glioma; KPS, karnofsky performance status; EOR, extent of resection; RTV, residual tumor volume; GTR, gross total resection.

**Table 2:** iUS and iMRI findings.

No	Pathology (WHO Grade)	Volume[cm^3^]	Number of iUS scan	Number of intermediate iUS scan	Number of margins marked by sulcus	Residual in Final iUS	Residual in first iMRI	Number of iMRI scans	Scenario for intermediated resection control *	Intraoperative decision making#
1	Glioblastoma (III-IV)	26.93	3	1	4	No	Yes	2	c	//
2	Glioblastoma (IV)	56.80	2	0	2	No	No	1	//	//
3	Glioblastoma (IV)	21.04	3	1	5	No	No	1	d	(2)
4	Glioblastoma (IV)	48.8	4	2	0	No	No	1	b	//
5	Glioblastoma (IV)	27.18	2	0	3	No	No	1	//	//
6	Anaplastic oligodendroglioma (III) (III)	177.42	3	1	0	No	No	1	a, d	(3)
7	Glioblastoma (IV)	57.43	2	0	3	No	No	1	//	//
8	Glioblastoma (IV)	145	2	0	3	No	No	1	//	//
9	Glioblastoma (IV)	110.95	3	1	4	Yes	Yes	1	a, b	(4)
10	oligodendroglioma (II)	43.94	5	3	5	Yes	Yes	2	a	(4)
11	oligodendroglioma (II)	24.73	3	1	5	Yes	Yes	1	a	(1)
12	Glioblastoma (IV)	92.96	4	2	2	No	Yes	1	a	//
13	Glioblastoma (IV)	69.63	2	0	5	No	No	1	//	//
14	Anaplastic astrocytoma (III)	93.5	3	1	3	Yes	Yes	1	a, c	(2)
15	Anaplastic oligodendroglioma (III) (III)	55.45	4	2	4	No	Yes	2	a, c	(2)
16	Astrocytoma (II)	7.83	3	1	0	Yes	No	1	c	//
17	Glioblastoma (IV)	8.14	3	1	4	No	No	1	d	(2)
18	Glioblastoma (IV)	85	2	0	5	No	No	1	//	//
19	Glioblastoma (IV)	161	4	2	4	No	Yes	2	c	//
20	oligodendroglioma (III)	70.40	3	1	5	No	No	1	d	(2)
21	Glioblastoma (IV)	72.90	4	2	4	No	No	1	b	//
22	Glioblastoma (IV)	36.56	2	0	4	No	No	1	//	//
23	Anaplastic astrocytoma (III-IV)	91.99	3	1	4	No	No	1	d	(2)
24	astrocytoma (II)	108	4	2	4	No	No	1	a	(1)
25	oligodendroglioma (II)	37.95	4	2	5	Yes	Yes	1	a	//
26	astrocytoma (II)	23.49	4	2	4	No	No	1	d	(3)
27	Glioblastoma (IV)	20.00	2	0	3	No	No	1	//	//
28	Anaplastic astrocytoma (III)	47.82	3	1	4	No	No	1	d	(3)
29	Glioblastoma (IV)	48.43	2	0	3	No	No	1	//	//
30	Glioblastoma (IV)	100.78	3	1	3	No	No	1	a	//
31	oligodendroglioma (II)	89.20	4	2	4	No	Yes	2	a	//
32	Astrocytoma (IV)	40.45	2	0	0	No	Yes	2	//	//
33	Glioblastoma (IV)	7.39	2	0	0	No	No	1	//	//
34	Glioblastoma (IV)	8.00	2	0	0	No	No	1	//	//
35	Glioblastoma (IV)	10.79	4	2	0	No	Yes	2	a, b	//
36	Astrocytoma (II)	5.09	3	1	0	Yes	Yes	1	a	//
37	Astrocytoma (II)	148.52	5	3	2	No	No	1	a	(1)
38	Glioblastoma (IV)	57.64	2	0	4	No	No	1	//	//
39	Glioblastoma (IV)	54.40	5	3	0	No	Yes	1	a	(2)
40	Glioblastoma (IV)	78.60	6	4	0	Yes	Yes	1	a	//
		61.64 ± 44.53	3.15 ± 1.05	1.15 ± 1.05	3.8 ± 0.92	8 (20%)	15 (37.5%)	1.18 ± 0.38	27 (67.5%)	15 (37.5%)

iUS, intraoperative ultrasound; iMRI, intraoperative MRI.

* Scenarios for intermediated resection control.

a: Portions of the tumor adjacent to eloquent structures were resected gradually under intensive iUS resection control and electrophysiological monitoring.

b: The tumors which involved multiple lobes or grew into both hemispheres, were resected separately.

c: The tumor is irregularly shaped, or, multicentric.

d: It was hard to distinguish tumor from normal tissue under the microscope, At the same time, anatomical markers lacked.

# Intraoperative decision-making.

(1) The relationship between the tumor and eloquent structures changed during the resection process.

(2) Hyperechoic area mismatch with the enhanced area or High T2 signal area on MRI.

(3) The glioma was diffuse, lacking definite margins in MRI images.

(4) The distance to the eloquent area on iUS didn’t agree with physiological monitor results.

### Clinical characteristics

There were 22 women and 18 men, with a mean age of 48.28 ± 12.66 years. The mean preoperative KPS was 90.25 ± 12.08. The pathological results revealed a total of nine (22.5%) LGGs (WHO grade I–II) and 31 (77.5%) HGGs (WHO grade III-IV). Twenty-five (62.5%) tumors were located in the regions surrounding the eloquent areas (motor or language areas). The mean tumor volume was 61.64 ± 44.53 cm^3^.

### EOR, GTR rate, and final residual tumor volume

The mean EOR was 95.43 ± 10.37%. The GTR rate was 72.5% (29/40), and the median residual tumor volume was 6.39 cm^3^ (range 1.06–16.23 cm^3^). Considering only the HGGs, the mean EOR was 97.34 ± 5.37%, the GTR rate was 77.4% (24/31). As for the LGGs, the mean EOR was 88.83 ± 18.78%, the GTR rate was 55.56%.

### Complications and functional outcome

No intraoperative complications occurred. In 57.5% (23/40) of patients, the postoperative neurological function was the same as or better than preoperatively. 42.5% (17/40) of patients had newly developed neurological deficits postoperatively; fifteen patients recovered from having neurological function equal to or better than their preoperative status during follow-up after surgery, while 5% (2/40) of patients had permanent deficits. At the final follow-up, the mean KPS was 85.0 ± 23.7 (range 30–100). Two elder patients developed severe respiratory complications at home, and one patient developed cerebral infarction two weeks after surgery. These three patients’ KPS scores decreased obviously after surgery, which lead to lower KPS scores after surgery.

### iMRI findings

The first iMRI scan detected residual tumors in 37.5% (15/40) of patients. Based on the first iMRI scan results, the EOR was 93.63 ± 11.32%.

17.5% (7/40) of patients with residual tumors detected on the first iMRI scan received further resection. The other eight patients did not receive further resections because of changes in neurophysiological monitoring. Finally, 10% (4/40) more patients achieved GTR. The other three of seven patients had small residual tumors left for function protection.

The number of iMRI scans per patient was 1.18 ± 0.38. 17.5% (7/40) of patients received two iMRI scans. No patients received more than two iMRI scans. The surgical time was 4.5 ± 3.6 hours.

### iUS findings

In 75% (30/40) of patients, a sulcus was very close to at least one of the tumor borders and could be regarded as the marker of the tumor margins. An average of 3.8 ± 0.92 tumor borders could be marked by a sulcus.

In 67.5% (27/40) of patients, the intermediate resection control was utilized to monitor the resection process. In these cases, 59.2% (16/27) had lesions adjacent to eloquent structures requiring gradual resection under intensive iUS resection control and electrophysiological monitoring. 14.8% (4/27) had large and multi-lobular tumors. 18.5% (5/27) had an irregularly shaped or multicentric tumor. 25% (7/27) had tumors that were difficult to distinguish from normal tissue under the microscope. The average number of iUS scans per patient was 3.15 ± 1.05. The time required for ultrasound image acquisition was 2-5minutes.

In 37.5% (15/40) of patients, intraoperative decisions were made to change the surgical procedures based on the iUS findings. In these cases, 46.67% (7/15) were because of a mismatch between iUS and MRI, 20% (3/15) were because the relationship between the tumor and eloquent structures changed during the resection process, 20% (3/15) were because the glioma was diffuse and lacked definite margins on MR images, 13% (2/15) were because the distance to the eloquent area on iUS did not agree with the physiological monitoring results.

Compared with the iMRI results, the sensitivity and specificity of iUS for residual tumors were 46% and 96%, respectively.

## Discussion

In this study, 40 glioma patients who underwent initial surgery at our center were reviewed retrospectively. We utilized the full-course resection control strategy by combining iUS and iMRI during surgeries. The final surgical results were good and comparable with the literature data. We found this strategy might have the following advantages (1): Improving the stability of the resection control (2); Providing more information during the pre-resection phase and intermediate stage (3); Enhancing the surgeon’s ability to modify surgical procedures during surgery (4). Compared with iMRI-only surgeries, the surgery time did not increase. Based on our data, this strategy was feasible and beneficial for glioma surgeries. However, there are also a few limitations that need to be mentioned.

### The surgical results and comparison with the literature data

In this study, the final EOR was 95.43%, and the GTR rate was 72.5%. 42.5% of patients had transient neurological deficits, and 5% were permanent. For comparison, **Table 3** lists the surgical results of some typical studies. Bastos’s study also combined 3D iUS and iMRI during surgeries (19). Their results were similar to ours. Scherer et al. (7) and Ghinda et al. (15) reported two large iMRI case series. Tuleeasca et al. conducted a meta-analysis using the latest iMRI data (26). In the studies using iUS, Shetty et al (23) and Munkvold et al (24) recruited large, unselected cohorts of gliomas at neurological centers with extensive iUS experience. Mahboob’s meta-analysis was based on fifteen iUS studies involving 739 glioma patients (25). The proportion of HGG, eloquent localism, and tumor volume of our cohort were comparable to those of large unselected cohorts reported by Shetty et al. (23) and Sheerer et al. (7). These variables have been proved to be important factors affecting EOR during glioma surgeries (7). In this respect, our surgical results were comparable with those reported in the literature, also in accordance with the meta-analysis.

**Table 3:** Surgical results of the typical studies.

Study	Patients(n)	Tumor volume[cm^3^] mean (SD)	Proportion of HGGn (%)	Eloquent location n (%)	Imaging Modality	EOR	GTRn (%)	Transient deficits n (%)	Permanent deficits n (%)
This study	40	61.64 (44.53)	31 (77.5%)	25 (62.5%)	iUS Navigation iMRI	95.43%	29 (72.5%)	17 (42.5%)	2 (5%)
Bastos (19)	23	//	8 (34.7%)	15 (65%)	iUS Navigation iMRI	//	12 (53%)	5 (21%)	0
Scherer (7)	224	31.27 (1.98)	180 (80.8%)	64 (28.6%)	Navigation iMRI	//	151 (67.4%)	30 (13.4%)	15 (6.7%)
Ghinda (15)	106	58.0 (37.9)	42 (39.6%)	106 (100%)	Navigation iMRI	92%	64(60.4%)	48 (46.2%)	9 (8.7%)
Tuleasca (2020)	527	//	//	//	Navigation iMRI	53%-100%	56.3% (47.5-65.1%) *	27.4% (15.2–39.6%) *	4.1% (1.3–6.9%) *
Shetty (23)	210	//	174 (83%)	156 (75%)	iUS Navigation	//	123 (58.6%)	//	35 (16.8%)
Munkvold (24)	144	//	97 (67%)	//	iUS Navigation	//	39 (27%)	//	//
Mahboob (25)	739	//	//	//	iUS Navigation		77% (67.1-86.9%) *	//	11.3%

iUS, intraoperative ultrasound; iMRI, intraoperative MRI; LGG, low-grade glioma; EOR, extent of resection; GTR, gross total resection; * 95% confidence interval.

We thought the results were good for these patients. For the LGGs, the mean EOR was 88.83 ± 18.78%, and the GTR rate was 55.56%. ≥ 80% EOR was achieved in 89% of the patients. Patients with LGGs resected ≥ 80% could have a significant survival advantage (2, 5). Considering the HGGs, the mean EOR was close to 97%. 77.4% of patients with HGG achieved GTR safely. The beneficial cut-off value was not consistent for the HGG. Lacroix et al. reported that over 93% EOR has a significant survival advantage (3). Sanai et al. showed that over 78% EOR could have a significant survival advantage (1). Oppenlander et al. reported 80% EOR can improve OS (27). Even if EOR exceeds 90%, every 5% increase can still significantly improve OS (1). Our results should have survival advantages for this group of patients. Because of the retrospective nature of our study design, we cannot conclude that combining iUS and iMRI can achieve better results compared to iUS or iMRI alone. As we have observed, the benefits of this strategy were manifested in other ways.

### Stabilizing the quality of the resection control

This study suggested that combining iUS and iMRI might make the quality of resection control more stable during glioma surgery. There was a wide variation in the reported results of glioma surgeries in the literature, no matter whether iUS or iMRI was used (20, 25, 26). For example, very different GTR rates were reported in the iMRI series: Nimsky et al. 31% (28), Maldaun et al. 40.5% (29), Ghinda et al. 60.4% (15), Senft et al. 96% (30). Mostly, this variability was due to the heterogeneous nature of gliomas (20). Therefore, maintaining resection control stability in different kinds of gliomas is equally important to improving its quality.

For the iUS, image quality had been confirmed as independent factor associated with GTR. There was also association between accuracy of the resection process assessment and ultrasound image quality (20, 24). However, The quality of iUS image is affected not only by operator experience but also by a variety of other factors, such as the recurrent status, extensive surrounding edema, radiation therapy, deep-seated location, large resection cavity, and bleeding (19, 21). The artifact of iUS also becomes apparent with the resection process (16). Therefore, it is difficult to fully control the quality of iUS images because of so many influencing factors. Even in centers with extensive iUS experiences, poor image quality was observed in 26% of a large, unselected cohort (20). Our data were similar regarding the iUS image quality. In 20% (8/40) of patients, iUS image quality wasn’t good enough to reveal the residual tumor found by iMRI. In this regard, combining iMRI might effectively compensate for the unstable image quality of iUS and maintain the quality of resection control. This is supported by our data: 10% more patients achieved GTR and EOR increased from 93.63% to 95.43% when iMRI was used for final control.

Conversely, iUS may compensate for the limitations of iMRI in other kinds of situations. In comparison to MRI, iUS has a higher sensitivity for detecting tumor margins in the initial assessment(77% vs. 69% for HGG (17), and 74% vs. 59% for LGG (16)). During resection, iUS could retain a good sensitivity (87%) (18). This allows it to monitor the tumor resection process from the beginning instead of just performing the final control as iMRI does (11, 31). As a result of the different ways of resection-control, diverse ranges of applications can be derived. In a multivariable analysis of iMRI, tumor volume, pathologic results, recurrence, and eloquent location were significant predictors of further resection in glioma surgeries (7). While for the iUS, the aim of total resection, single tumor, image quality, and eloquent location were factors predicting GTR (20). The tumor volume and pathological results were insignificant during multivariable analysis (20). In gliomas with large volumes or some type of glioma with atypical MRI appearances, iUS might compensate for the limitations of iMRI. This was supported by the observations from this study.

For very larger tumors, iUS could help us identify the compressed anatomical structures not visible in MRI images, establish clear resection limits at the beginning of the resection, and control the resection process following these limitations (Case 1). We achieved good surgical results safely, and with lesser iMRI scans. In 25% (12/40) of the patients, the tumors’ volume was larger than 85cm^3^. The number of iUS scans was on average 3.3. Three (25%) unexpected residual tumors were confirmed by iMRI. Two (16%) received further resection and two iMRI scans. None had newly permanent deficits after surgery. In comparison, Bohinski et al (32) reported that 7(63%) patients needed further resection in 11 patients with glioma greater than 70cm^3^. Scherer et al (7) reported that 69% of patients needed further resection in a group of patients with an average of 31.27 ± 1.98 cm^3^ gliomas. The long-term deficits were 6.7%.

In 33% (10/40) of the patients, the tumors had atypical MRI appearances. Some of them were diffused, lacking clear boundaries on MRI images (case 3). Lesions with diffuse boundaries on MRI are not also diffuse in ultrasound images (20). Moiyadi et al. reported that 7 had relatively clear margins in 10 gliomas with diffused margins on MRI (10). Others demonstrated a mismatch with iUS images (case 2). Hartov et al. also reported that agreement between the two imaging modalities was observed in 40% of cases (33). In these situations, it was hard to establish clear resection limits using MRI images in these patients. It was also challenging to control the resection using iMRI. At these times, IUS was greatly helpful in establishing the resection limits (10), maximizing tumor cell removal (Case 2), while balancing EOR and protection of functions (Case 3).

### Providing more information during the pre-resection and intermediate stages

In 75% (30/40) of patients, we observed that an average of 3.8 borders of the tumor were beside the sulcus during the pre-resection iUS scan. Due to resolution issues and partial volume effects, preoperative MR images could not clearly depict these small sulci. By contrast, they could be visualized in iUS images, even when compressed by the tumor (**Figure 2C**) (19). During surgery, these sulci could serve as landmarks to identify the tumor margins, and resection limits. We found this maneuver to be very useful during surgery because of the following reasons (1): These sulci could remind the surgeon where to start or stop the resection process accurately, especially when it was hard to distinguish the tumor from normal brain tissue under the microscope, or evident brain shift happened. According to literature data, over 50% of patients had an average of 8 mm of brain shift just after the dural opening, which would be more evident with the resection process (34) (2). Compared with the pointer of the navigation system, the large contact surface of the iUS probe might lead to the loss of the target after moving the probe away from the surgical area. Using the constant sulci as landmarks, the surgeon could quickly identify the position changes of the tumor boundaries, even when a noticeable brain shift occurred.

In 32.5% of the patients, the intermediated iUS scan was not used. These tumors always had regular shapes and clear boundaries under the microscope. Additionally, they were away from the eloquent structures. Experienced surgeons could perform perfect resections without the help of intraoperative imaging. It was also approved by the iMRI results: the residual tumors were not detected in these patients without receiving intermediate scans. Nevertheless, such tumors constituted the minority of daily gliomas. In most of the patients (67.5%), the intermediate resection control was utilized to monitor the resection process. Uncertainties about the resection process existed in most of the surgeries, even for very experienced surgeons. The intermediate iUS scans were useful in helping the surgeon get additional information, resolving the uncertainties, and modifying the surgical strategies.

**Figure 4:**
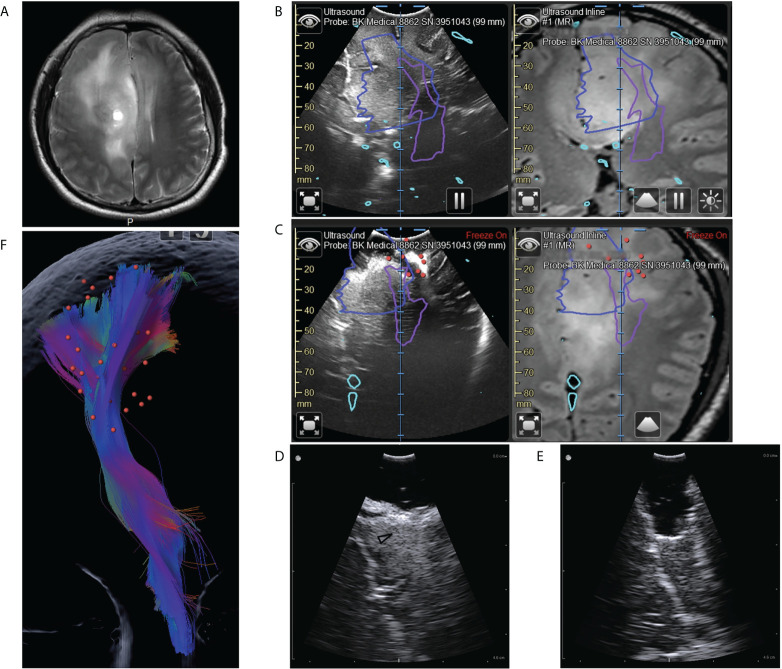
Illustrative case 3. **(A)** On the preoperative MRI images, a tumor could be seen in the right frontal lobe. It was diffuse and lacked defined boundaries. **(B)** Coplanar MRI and iUS images showed the hyperechoic area was much smaller than the area with abnormal signals on flare images (blue line). Purple lines represented the pyramidal tract (PT) boundaries revealed by DTI. **(C)** Intermediate iUS images showing the resection close to the PT. In accordance with iUS, we purposefully increased the spatial and temporal intensity of mapping. The red dots indicate areas where we got positive reactions at the bottom of the resection cavity. The lowest current intensity was 3mA. According to the mapping result, we should stop resection. However, the iUS images revealed a tumor on the medial side of the PT (white arrow). **(D, E)** The residual tumors (black arrowhead) were then removed with the help of a small bur hole transducer. **(F)** The spatial relation between the positive mapping sites (red dots) and the pyramidal tract (color streamlines). By integrating iUS with navigation, we were able to position mapping points accurately and comprehensively.

In 40% (16/40) of patients, intermediate iUS scans were used to assist the mapping technique when approaching the eloquent areas (Case 3). An issue with the mapping technique is that inaccurate spatial and temporal intensity might cause unexpected mechanical damage to the eloquent area (35). Utilizing iUS, the distance and relative position to the eloquent structure can be depicted clearly, which allows for optimizing the spatial and temporal intensity of mapping, assuring the accuracy of the mapping process and reducing the risk of unexpected damage. As shown in case 3, we purposefully increased the intensity of the mapping as iUS reminded us that we were close to the pyramidal tracts (**Figure 4C**). Using the combined images of iUS and DTI, we were able to identify the safe boundaries around PT with greater accuracy (**Figure 4F**). In addition, we could determine the shape of the tumor distribution surrounding the PT (**Figure 4C**), which was not possible with only mapping. The information allowed us to remove as much tumor tissue as possible while preserving function (Case 3).

### Enhancing the ability to modify surgical strategy intraoperatively.

In the literature, the intraoperative decisions were mainly about whether further resections were needed. Further resection rates ranged from 26.1%-52.2% for the iMRI series and 25.8%-42.5% for the iUS series (23, 36). The present study showed that intraoperative decisions were not limited to further resection. In addition to 17.5% (7/40) further resections based on iMRI results, the surgical procedures in 37.5% (15/40) of the patients were modified based on iUS findings. As a result, 2-fold more intraoperative decisions were made to tailor the surgical strategy, compared with the need for further resection.

In 17.5% (7/40) of patients, we observed a mismatch between tumor borders on iUS and preoperative MR images (Case 2, 3). We modified resection boundaries for these patients based on iUS findings because iUS had higher sensitivity for detecting glioma margins (16, 17, 37, 38). In 7.5% (3/40) of patients, iUS after partial resection of the tumors revealed relationships between the tumors and eloquent structures, which differed from our preoperative assessment. Therefore, surgical plans were changed accordingly (Case 1). In other 7.5% (3/40) of the patients, the tumors were diffuse and lacked definite boundaries (Case 3). Resections were performed according to the iUS, which showed more precise boundaries. In 5% (2/40) of patients, we identified obvious residual tumors, while the mapping had reached the threshold of 3-5mA (Case 3). We performed further resection according to the iUS findings, without leading to permanent deficits. These experiences showed that combining iUS and iMRI could provide surgeons with more intraoperative information, thus allowing for more informed and, thus, higher-quality decisions.

### Improved surgical efficiency

Although introducing iUS to this group of patients might not impact EOR and GTR rates, the number of iMRI scans was significantly reduced. The mean number of iMRI scans was 1.18 per patient, and only 17.5% of patients received two iMRI scans. In comparison, previous studies using the same iMRI system report an average of 1.8 iMRI scans (two to six scans per patient in over 48% of patients) (13, 14). The reduction of iMRI scans was due to fewer patients requiring further resections. Further resection was recorded in 17.5% of patients, compared to 46% in the previous iMRI case series (13–15, 26). One iMRI scan session needs about 30–70 minutes when using the high field iMRI system (13–15, 39). Reducing the number of iMRI scans should effectively shorten the surgery time.

In this study, we observed a reduction in surgery time compared with iMRI-assisted surgery. The average surgical time was 4.5 hours in this group of patients. Leuthardt et al. reported that the surgical time using iMRI was average 7.9 hours (40). Maldaun et al. reported average 7.3 hours (29). Lu et al. reported average 5.91 hours (41). Hamilton et al. reported average 6.1 hours (42). Many studies showed that iMRI-guided surgery was time-consuming (43). Patient selection and schedules for the surgeon and operating room staff can be impacted by the prolonged operative time of iMRI-assisted surgeries (43). This is one reason for the selected patient populations in the iMRI series (13, 14, 28). A reduction of surgery time should improve the efficiency of iMRI-assisted surgery and reduce the risk of adverse events associated with long surgery times.

### Limitations

There were some apparent limitations in implementing this strategy. One is the cost of the system. The iMRI, iUS, and navigation systems are extremely expensive. Only a few large neurosurgical centers can afford them. Another limitation stems from the steep learning curve associated with iUS, iMRI, and navigation systems. Adequate training and experience are obliged for successfully utilizing these techniques during surgery.

Moreover, combining them efficiently and reasonably is also challenging, even for surgeons at the right learning curve stage. These financial and education costs will significantly limit the implementation of this strategy. However, iMRI has been more prevalent worldwide over the past few years. iUS systems are becoming smaller and more portable. As a result of these trends, this strategy is more likely to become viable in the near future.

This study also has some limitations that must be addressed. This was a retrospective study based on the data from one neurosurgical center, and therefore selection bias was inevitable. The recurrent cases were not included. The sample size was also limited, especially for patients with LGG. Due to equipment limitations, we did not use other advanced iUS techniques such as elastography or contrast-enhanced ultrasound in this study. These techniques might result in different surgical results if combined with iMRI. Further studies are necessary regarding the usefulness of combining advanced iUS techniques and iMRI in a large unselected glioma cohort.

## Illustrative cases

### Case 1 (patient #37)

The patient was a 40-year-old female without symptoms. The MRI showed a large glioma in the right temporal lobe without enhancement (**Figure 2A**). The tumor volume was 148.52 cm^3^. On preoperative MRI images, anatomical structures were compressed by the large tumor and were difficult to identify. Some critical information for the surgical strategy could not be obtained from MRI images: 1. The tumor’s relationship with the insular lobe and the Sylvian fissure. 2. The location of the temporal horn of the lateral ventricle. 3. was the tumor growing into the basal ganglia through the temporal stem? On the pre-resection iUS images, the compressed insular lobe and Sylvian fissure could be clearly seen (**Figure 2B**). We found the tumor isolated from the Sylvian fissure by a thin slice of normal tissue and not associated with the insular cortex. Hence, the Sylvian fissure was used as the upper limit of resection. On the pre-resection iUS, a small sulcus was also seen just alongside the tumor’s posterior border (**Figure 2C**). We used this sulcus as the posterior limit for the resection. The middle skull base was intended to be the anterior and low limit of resection. This way, clear resection limits could be established according to the iUS images. We used a serial of iUS scans to monitor the resection and paid close attention to the relationship between the tumor and basal ganglia. Following the removal of the majority of the tumor, the compressed ventricle and temporal stem became evident (**Figure 2E**). We noticed that the tumor did not grow into basal ganglion along the temporal stem. The information was crucial to the following surgical strategy. We were able to remove the tumor along the temporal stem without worrying about damage to the pyramidal tract. The mapping procedure prepared in advance was not used during surgery. The iMRI images confirmed that the tumor had been removed entirely along with the planned resection limits and temporal stem (**Figure 2F**). No residual tumor was founded. After surgery, the patient had no neurological deficits.

### Case 2 (patient #17)

The patient was a 60-years-old female who had suffered from headaches for two months. MRI showed a lesion with obvious enhancement at the right frontal lobe. There was a large area with long T1 and long T2 signals around the enhanced lesion, which might result from edema. Our initial surgical plan was to remove the enhanced lesion. Utilizing the navigation system, we registered iUS with MRI during surgery. On the coplanar images of iUS and MRI, we noticed that the signal intensity in the suspected edema area was similar to that in the enhanced area and much higher than the normal tissue (**Figures 3A** **, B**). Based on this unusual finding, we modified our surgical plan. 3D iUS volume and fast pathological examination were used to guide the resection process (**Figures 3C** **, E, G**). During surgery, we obtained tissue samples within the enhanced area. At this site, iUS had the highest signal (**Figure 3C**). Fast-frozen pathological examination revealed HGG, which was confirmed by the final pathological examination (**Figure 3D**). The second tissue sample was taken outside the enhanced lesion, where MRI showed an abnormally long T1 and long T2 signal. Co-planar 3D iUS showed a similar signal at this site as the enhanced part (**Figure 3E**). The fast-pathological examination still showed obvious infiltration of tumor cells, confirmed by the final pathological examination (**Figure 3F**). So, we removed these tissues continuously, guided by 3d iUS (**Figure 3E**). After that, the third sample was taken after all tissue with abnormal MRI signals had been removed. At this site, the iUS were still hyperechoic, but slightly lower than at the previous site (**Figure 3G**). Tumor cells were still found in the pathological results (**Figure 3H**). At last, all the hyperechoic area on the iUS was removed. Final pathology results reveal glioblastoma (WHO grade IV). The volumetric analysis of resected tissue according to iUS calculated 60.6cm^3^ (**Figure 3I**). The volume of the enhanced lesion was 8.1cm^3^, equal to only 13.4% of the resection volume (**Figure 3J**). The tumor did not relapse after one year of follow-up.

### Case 3 (patient #28)

The patient was 40 years old male with the symptom of epilepsy. MRI showed a lesion occupying the right frontal lobe without noticeable enhancement. On the T2 images, the lesion was diffused, lacking clear boundaries (**Figure 4A**). It was hard to establish the resection limits using MRI images. DTI and fiber tracking results showed that the pyramidal tract passes through the area with a hyper T2 signal (**Figure 4B**). It was also challenging to balance EOR and function protection for this patient. Utilizing the navigation system, we registered iUS with MRI during surgery. On the coplanar images of iUS and MRI, we noticed that the hyperechoic area was much smaller than the area with abnormal signals on flare images (**Figure 4B**). According to this finding, We, therefore, decided to perform a resection according to iUS and fast pathological examination. During surgery, we found that the hyperechoic tissue was more rigid compared with the surrounding tissue. The fast-pathological examination confirmed that the hyperechoic tissue was LGG, whereas the hypoechoic tissue with a high T2 signal was not tumor tissue. A serial of iUS cans were used to monitor the distances to the pyramidal tract as we approached the pyramidal tract (**Figure 4C**). According to iUS, we purposefully increased the spatial and temporal intensity of mapping surrounding the pyramidal tract (**Figure 4F**). As the current intensity was as low as 3mA, resection was stopped (**Figure 4C**). iUS showed that the resection cavity was just beside the pyramidal tract, but there was still a residual tumor at the midline side of the pyramidal tract (**Figure 4C**). We further removed this part of the tumor and used the bur hole transducer to control the resection process (**Figures 4D** **, E**). In the end, all the hyperechoic tumors were moved successfully. The final pathological examination revealed oligodendroglioma (WHO III). After surgery, the muscle strength of the patient was grade 1 and recovered to grade 5 seven days after surgery.

## Conclusion

The full-course resection control strategy by combining iUS and iMRI could be successfully implemented with good surgical results in initial glioma surgeries. This strategy might stabilize resection control quality, and provide the surgeon with more intraoperative information to tailor the surgical strategy. In comparison with iMRI-assisted glioma surgery, this strategy might improve efficiency, by reducing the number of iMRI scans and shortening surgery time.

## Data availability statement

The original contributions presented in the study are included in the article/Supplementary Material. Further inquiries can be directed to the corresponding author.

## Ethics statement

The studies involving human participants were reviewed and approved by Ethics Committee of Xuanwu Hospital Capital Medical University. The patients/participants provided their written informed consent to participate in this study. Written informed consent was obtained from the individual(s) for the publication of any potentially identifiable images or data included in this article.

## Author contributions

Conceptualization, YH, JT Methodology, YH, JT. Validation, YH, YL. Investigation, YH, YL. Resources, JT, QL, YY. Data processing, YH, YL, QL, YY. Writing—original draft preparation, YH. Writing—review and editing, JT. Visualization, YL, QL, YY. Supervision, YH, JT. Project administration, YH, JT. All authors contributed to the article and approved the submitted version.

## Conflict of interest

The authors declare that the research was conducted in the absence of any commercial or financial relationships that could be construed as a potential conflict of interest.

## Publisher’s note

All claims expressed in this article are solely those of the authors and do not necessarily represent those of their affiliated organizations, or those of the publisher, the editors and the reviewers. Any product that may be evaluated in this article, or claim that may be made by its manufacturer, is not guaranteed or endorsed by the publisher.
